# STAT3 Drives GFAP Accumulation and Astrocyte Pathology in a Mouse Model of Alexander Disease

**DOI:** 10.3390/cells12070978

**Published:** 2023-03-23

**Authors:** Tracy L. Hagemann, Sierra Coyne, Alder Levin, Liqun Wang, Mel B. Feany, Albee Messing

**Affiliations:** 1 Waisman Center, University of Wisconsin-Madison, Madison, WI 53705, USA; scoyne4@wisc.edu (S.C.); alevin5@wisc.edu (A.L.); amessing@wisc.edu (A.M.); 2 Wyss Institute, Harvard University, Boston, MA 02115, USA; liqun.wang@wyss.harvard.edu; 3 Department of Pathology, Brigham and Women’s Hospital, Harvard Medical School, Boston, MA 02115, USA; mel_feany@hms.harvard.edu; 4 Department of Comparative Biosciences, School of Veterinary Medicine, University of Wisconsin-Madison, Madison, WI 53705, USA

**Keywords:** GFAP, STAT3, astrocyte, Alexander disease, Rosenthal fibers, reactive gliosis

## Abstract

Alexander disease (AxD) is caused by mutations in the gene for glial fibrillary acidic protein (GFAP), an intermediate filament expressed by astrocytes in the central nervous system. AxD-associated mutations cause GFAP aggregation and astrogliosis, and GFAP is elevated with the astrocyte stress response, exacerbating mutant protein toxicity. Studies in mouse models suggest disease severity is tied to *Gfap* expression levels, and signal transducer and activator of transcription (STAT)-3 regulates *Gfap* during astrocyte development and in response to injury and is activated in astrocytes in rodent models of AxD. In this report, we show that STAT3 is also activated in the human disease. To determine whether STAT3 contributes to GFAP elevation, we used a combination of genetic approaches to knockout or reduce STAT3 activation in AxD mouse models. Conditional knockout of *Stat3* in cells expressing *Gfap* reduced *Gfap* transactivation and prevented protein accumulation. Astrocyte-specific *Stat3* knockout in adult mice with existing pathology reversed GFAP accumulation and aggregation. Preventing STAT3 activation reduced markers of reactive astrocytes, stress-related transcripts, and microglial activation, regardless of disease stage or genetic knockout approach. These results suggest that pharmacological inhibition of STAT3 could potentially reduce GFAP toxicity and provide a therapeutic benefit in patients with AxD.

## 1. Introduction

Alexander disease (AxD) is a progressive and generally fatal disorder of the central nervous system (CNS), with a range of clinical phenotypes including cognitive and motor impairment and white matter deficits that are distributed throughout the neuroaxis [1]. AxD has been subclassified into two groups, with type I demonstrating frontal predominance and early onset, and type II presenting with lesions concentrated in the hindbrain and cervical spinal cord, less pronounced leukodystrophy and cognitive deficits, and onset at any age [2]. The hallmark pathological feature in both groups is the presence of Rosenthal fibers, cytoplasmic protein aggregates of astrocytes, particularly in perivascular, subpial, and subependymal locations.

Nearly all cases of AxD result from heterozygous missense mutations in the astrocyte intermediate filament, GFAP [3,4]. To better understand how mutations in GFAP cause disease, we generated mouse models with knock-in mutations causing single amino acid changes orthologous to common human mutations at the endogenous *Gfap* locus [5]. These models replicate many of the pathological characteristics found in the human disease. AxD mice spontaneously increase expression of *Gfap* above normal levels, form the characteristic Rosenthal fibers within the cytoplasm of astrocytes, induce stress responses including Nrf2/ARE and small heat shock protein pathways, and activate microglia [6].

A major factor in the pathology induced by mutant GFAP in AxD is accumulation of GFAP protein to toxic levels. Even overexpression of wild-type GFAP causes protein aggregation, and at high enough levels, is lethal [7]. The cascade of stress response signaling pathways associated with protein misfolding leads to reactive gliosis with increased GFAP expression, which is in part due to transactivation of the *Gfap* promoter [8]. The transcription factor STAT3 regulates astrocyte development and astrogliosis and directly controls transcription of *Gfap* [9,10,11,12,13]. STAT3 binds to the *Gfap* promoter and is necessary to induce expression [13,14], and STAT3 activation has been shown to drive reactive gliosis in several models of acute injury [11,12,15] and neurodegeneration [16,17].

Although the disease process in AxD is initiated by expression of mutant protein, the resulting astrogliosis and transactivation of the *Gfap* promoter further promote protein accumulation and toxicity [8]. We have shown that STAT3 is phosphorylated in both mouse and rat models of AxD [18,19] and hypothesize that activation of STAT3 contributes to a positive feedback loop that drives increased GFAP expression, protein accumulation, and ultimately astrocyte dysfunction. Here, we used a combination of genetic approaches to manipulate STAT3 activation and clarify its involvement in chronic gliosis and to test whether blocking phosphorylation reduces astrocyte pathology in mouse models of the disease. Therapeutic approaches to target activation of the JAK/STAT pathway have been approved to treat inflammatory disorders, hematopoietic malignancies, and solid tumors [20,21], and inhibitors that cross the blood-brain barrier could potentially be repurposed to benefit patients with AxD.

## 2. Materials and Methods

### 2.1. Mice

All animal studies were approved by the College of Letters and Sciences and Vice Chancellor Office for Research and Graduate Education Animal Care and Use Committee within the University of Wisconsin-Madison. *Gfap*^+/R236H^ mice (*Gfap*^tm2Mes^) were maintained as heterozygotes congenic in either FVB/N-Tac or C57BL/6J genetic backgrounds [5]. *Stat3*^fl/fl^ mice (*Stat3*^tm2Aki^) [22], engineered with loxP sites flanking the exon coding for the Tyr-705 phosphorylation site, were congenic in C57BL/6J and maintained as homozygotes. Tg(Gfap-cre)73.12Mvs (C57BL/6J, JAX stock #012886) [23] and Tg(Aldh1l1-cre/ERT2)1Khakh (C57BL/6NJ, JAX stock #031008) [24] lines were maintained as hemizygotes. Ai14 Cre reporter mice (Gt(ROSA)26Sor^tm14(CAG-tdTomato)Hze^/J, C57BL/6J, JAX stock #007914) [25] were maintained as heterozygotes. Heterozygous constitutive *Stat3*^+/−^ knockout mice were generated by crossing a nestin-promotor-driven Cre line (Tg(Nes-cre)1Kln, C57BL/6J, JAX stock #003771) [26] with *Stat3*^fl/fl^ mice for germline excision of the floxed allele. Cre excision removes the exon containing the Tyr-705 phosphorylation site while leaving the remaining coding sequence in frame and the translated protein intact. The genes for STAT3 and GFAP are located within a 2 megabase interval on mouse chromosome 11 and required homologous recombination between the two genes to combine the floxed *Stat3* allele with the R236H *Gfap* mutation. These mice were maintained by crossing *Gfap*^+/R236H^; *Stat3*^fl/fl^ mice with *Stat3*^fl/fl^ mice. Genotyping was performed by Transnetyx using real time PCR to analyze tail biopsy DNA.

### 2.2. Human Brain Tissue

Frozen frontal cortex from a 1-year-old AxD patient was obtained from the NICHD Brain and Tissue Bank for Developmental Disorders at the University of Maryland, Baltimore, MD, USA. Tissue was thawed and fixed in 4% paraformaldehyde overnight before paraffin embedding for immunolabeling.

### 2.3. Tamoxifen Induction

For inducible Cre activation in mice expressing the *Aldh1l1*-CreER^T2^ transgene, all mice were given intraperitoneal injections of 80 mg/kg tamoxifen (Sigma T5648, dissolved in corn oil at 20 mg/mL) daily for 5 days starting at approximately P56. Personnel administering the injections were blinded to genotypes. Tissues were collected for molecular and histological analysis at 3 months of age (~P90) as described below.

### 2.4. Tissue Collection

For molecular analysis of crosses including *Gfap*^+/R236H^; *Stat3*^+/−^ or *Gfap*^+/R236H^; *Stat3*^−/fl^; *Gfap*-Cre^Tg73.12^ mice, animals were euthanized by CO_2_ asphyxiation, and tissues were collected on ice and frozen immediately with dry ice and stored at −80 °C until processed. For experiments performed with crosses to generate *Gfap*^+/R236H^; *Stat3*^fl/fl^; *Gfap*-Cre^Tg73.12^ mice, animals were first anesthetized with isoflurane and transcardially perfused with phosphate buffered saline (PBS, pH 7.4). Brains were collected and bisected at the midline: half was placed in 4% paraformaldehyde for histological analysis, and half was microdissected and frozen for molecular analysis.

For experiments performed with mice from the *Gfap*^+/R236H^; *Stat3*^fl/fl^; Aldh1l1-CreER^T2^; Ai14 cross, animals that were positive for Ai14 were anesthetized with isoflurane and transcardially perfused with phosphate buffer, followed by 4% paraformaldehyde for histological analysis. Animals that were Ai14 negative were euthanized by CO_2_ asphyxiation, and tissues were collected on ice and frozen immediately with dry ice and stored at −80 °C until processed.

### 2.5. RNA Extraction and Quantitative PCR

Tissues were homogenized in Trizol reagent (Thermo Fisher Scientific, Invitrogen, Waltham, MA, USA) for RNA analysis per the manufacturer’s protocol. For quantitative PCR, 1 µg RNA was transcribed as cDNA using 1 µm random hexamers, 2 µm poly-(dT)15 primers and 500 nm (each) dNTP in a 20 µL reaction with Superscript III (Thermo Fisher Scientific, Invitrogen protocol). Reverse transcriptase reactions were diluted and incorporated into quantitative PCR at a ratio of 16 nl cDNA per µL reaction using SYBR Green Master Mix with 150 nM primers (Table 1) on an ABI ViiA7 Real-Time PCR System (Thermo Fisher Scientific, Applied Biosystems Inc.). Standards generated from purified amplicons were used to determine relative concentrations, and values were normalized to *Rn18s*.

### 2.6. Protein Extraction and Western Analysis

Total protein lysates were prepared from frozen brain regions by homogenizing with a bead mill mixer (GenoGrinder, SPEX SamplePrep) with 50 mg tissue per ml 2% SDS, 50 mM, Tris-HCl (pH 7.4), 5 mM EDTA, 1 mM Pefabloc SC (Sigma-Aldrich, St. Louis, MO, USA) and Complete Protease Inhibitor Cocktail (Sigma-Aldrich, Roche). Samples were boiled for 20 min, and protein concentration was determined by bicinchoninic acid assay using the BCA Protein Assay kit (Thermo Fisher Scientific) with bovine serum albumin (BSA) as a standard. For western analysis, samples were diluted with lysis buffer to equivalent concentrations and 20–30 µg electrophoresed (125V at room temperature until bromophenol blue reached the bottom of the gel) on 26-well 10% Criterion Tris-HCl or TGX gels (BioRad, Hercules, CA, USA) before transferring to an Immobilon-FL membrane using the BioRad Criterion system. For most experiments, immunoblots were stained with REVERT Total Protein Stain (LI-COR Biosciences, Lincoln, NE, USA) and imaged with a LI-COR Odyssey for subsequent normalization (GAPDH was used for normalization otherwise). Membranes were blocked in SEA Blocking Buffer (Thermo Fisher Scientific, Pierce) before incubating with primary antibodies overnight at 4 °C. Immunoblots were washed before adding secondary antibodies and incubating for 2 h at room temperature, washing again, and imaging with a LI-COR Odyssey. Tris buffered saline (TBS, pH 7.5) with 0.05% Tween 20 was used for all antibody dilutions and washes. A final wash in PBS was performed before imaging. Antibodies are listed in Table 2.

### 2.7. GFAP ELISA

GFAP was quantified from total protein extracted with 2% SDS (described above) by a sandwich enzyme linked immunosorbent assay (ELISA) as described previously [8]. Briefly, microtiter plates (Nunc MaxiSorp, Thermo Fisher Scientific, Waltham, MA, USA) were coated with a cocktail of monoclonal GFAP antibodies diluted in PBS (SMI-26, see Table 2 for antibodies) and blocked with BLOTTO (5% milk in PBS) before adding protein samples and standards diluted in PBS with 1% BSA and 0.05% Tween 20 (Tw20). Plates were incubated at room temperature for 2 h and washed with PBS/0.05% Tw20 before adding rabbit anti-GFAP detection antibody (Dako Z0334) diluted in BLOTTO and incubated overnight at 4 °C. Plates were washed with PBS/0.05% Tw20 followed by incubation with secondary HRP conjugated goat anti-rabbit IgG diluted in BLOTTO at room temperature for 2 h. Plates were washed with PBS/0.05%Tw20 followed by PBS before adding SuperSignal ELISA Femto Substrate (Thermo Fisher Scientific, Pierce, Waltham, MA, USA) and analyzing with a GloRunner luminometer (Turner Biosystems).

### 2.8. Histological Analysis

Mouse brains were collected as described above, fixed in 4% paraformaldehyde overnight before cryoprotecting in graded concentrations of sucrose (10–30%), and sectioning on a sliding microtome at 40 µm. Sections were stored at −20 °C in 25% glycerol/25% ethylene glycol/0.1 M phosphate buffer (pH 7.4) as cryoprotectant. For labeling, tissue sections were rinsed with PBS, blocked and permeabilized with 5% normal goat or donkey serum, 0.5% Triton-X-100 (TX100) in PBS, and incubated in primary antibody (Table 2) diluted in 1% BSA, 0.3% TX100 in PBS for approximately 72 h at 4 °C. Sections were washed in PBS with 0.05% TX100 before incubating in secondary antibody (Table 2) diluted in 1% BSA, 0.3% TX100 in PBS overnight at 4 °C. Finally, sections were washed in PBS/0.05% TX100 and mounted with ProLong Gold mounting media with DAPI (Thermo Fisher Scientific, Invitrogen). For phophoY705-STAT3 immunofluorescence, sections were further permeabilized with 100% methanol for 10 min at −20 °C prior to adding block. Fluorescence images were collected with a Nikon A1R-HD confocal microscope system with equivalent settings for comparisons between genotypes.

### 2.9. Image Analysis

All fluorescent images were collected with identical settings at 1024 × 1024 pixel resolution for comparisons and quantification, and analysis performed on grayscale images from individual fluorescent channels. For signal intensity, the entire field of view was selected, and the mean pixel intensity measured with ImageJ/Fiji (NIH). Values are expressed as arbitrary units (a.u.) of fluorescence normalized to the control group. To quantify p62 as a representation of protein aggregation and Rosenthal fibers, gray-scale images were converted to RGB, and p62 positive puncta highlighted using the color threshold adjustment in Fiji (default thresholding method, dark background on) by setting the upper limit for brightness to separate high intensity signals from background in the histogram. The highlighted puncta were selected, and the measured area converted to a percentage of the total image area for comparisons.

## 3. Results

### 3.1. STAT3 Activation in AxD

**Figure 1:**
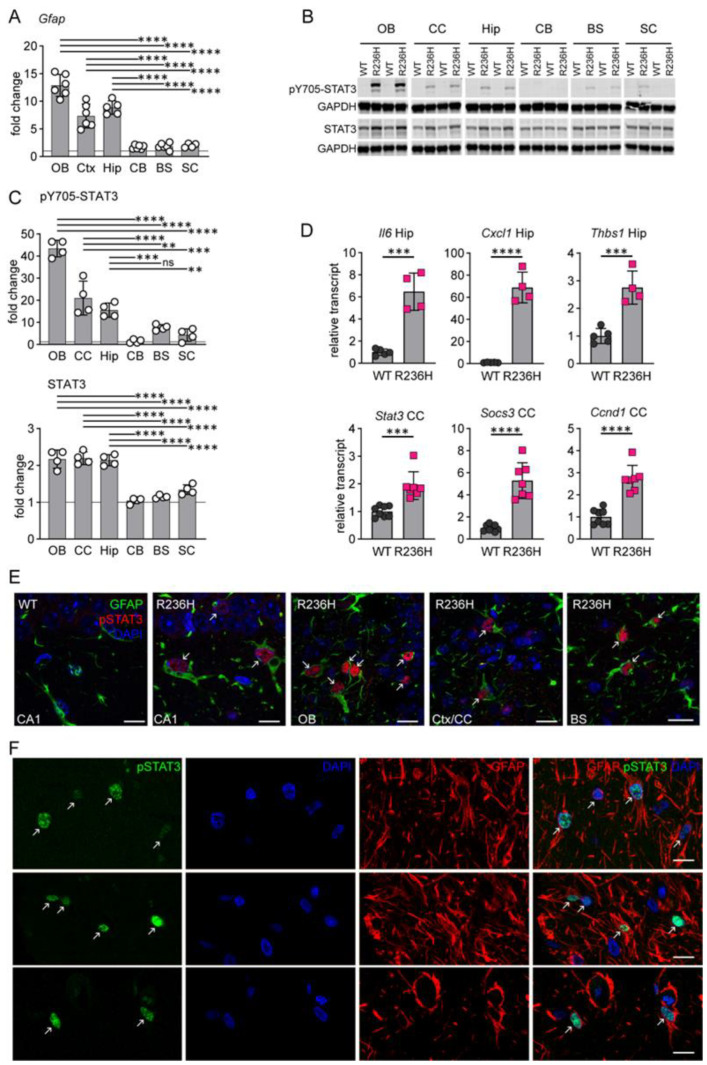
STAT3 activation in AxD. (**A**) Comparison of fold increase in *Gfap* transcript among different brain regions and spinal cord in *Gfap*^+/R236H^ mice as measured by qPCR. Results for olfactory bulb (OB), cortex (Ctx), hippocampus (Hip), cerebellum (CB), brainstem (BS) and cervical spinal cord (SC) are shown (males at 8 weeks, ANOVA with Sidak’s post-tests, data reanalyzed from Figure 2 of Jany et al., 2013 [8]). (**B**) Western analysis of pY705-STAT3 and total STAT3 in different brain regions and spinal cord of *Gfap*^+/R236H^ (R236H) and wild-type (WT) mice (different animal in each lane). (**C**) Graphs represent fold change comparisons of R236H over WT for immunoblots as shown in (**B**) (males at 3 months, ANOVA with Sidak’s post-tests) (**D**) Quantification of transcripts regulated by STAT3 or related to neuroinflammation in hippocampus (Hip) and corpus callosum (CC) in *Gfap*^+/R236H^ mice. Relative values are normalized to *Rn18s* and expressed as a fold change compared to the wild-type average (males and females, Hip at 8 weeks, CC at 12 weeks, two-tailed *t*-test). (**E**) Immunolabeling of pY705-STAT3 (red) and GFAP (green) in *Gfap*^+/R236H^ mouse hippocampus (CA1), olfactory bulb (OB), frontal cortex adjacent to the corpus callosum (Ctx/CC), and brainstem (BS). Left panel shows a wild-type (WT) mouse, and remaining panels represent *Gfap*^+/R236H^ mice (R236H, males and females at 2 months). (**F**) Immunolabeling of pY705-STAT3 (green) and GFAP (red) in gray matter astrocytes from a 1-year-old patient with AxD. Arrows in (**E**,**F**) indicate pSTAT3 labeled nuclei. All mice are FVB/N. ** *p* < 0.01, *** *p* < 0.001, **** *p* < 0.0001 for all statistics. Scale bars = 10 µm.

*Gfap*^+/R236H^ mouse models of AxD demonstrate GFAP accumulation and a marked astrocyte stress response. In addition to perivascular, periventricular, and subpial astrocytes, protein aggregation and increased GFAP expression occur predominantly in forebrain regions including hippocampus, corpus callosum and olfactory bulb [5,8]. As a marker of astrogliosis, changes in the *Gfap* transcript show a similar pattern with greater elevation in forebrain compared to hindbrain regions and spinal cord, as previously reported [8] (Figure 1A). STAT3 is known to transactivate *Gfap* expression in reactive gliosis [12,15], and transcriptome analysis of a mouse model of AxD overexpressing human GFAP also suggests JAK/STAT pathway activation [27]. To determine whether STAT3 activation correlates with GFAP elevation in mouse models of AxD, we performed western analysis of STAT3 and phosphorylated STAT3 (Tyr705) in different regions of the CNS and found that STAT3 expression and activation were more pronounced in forebrain regions compared to hindbrain regions and spinal cord, similarly to *Gfap* transcript (Figure 1B,C). Although STAT3 protein was elevated approximately 2-fold, expression levels did not account for the 15- to 40-fold increase in phospho-STAT3. Further analysis showed other genes regulated by STAT3, including *Il6*, *Socs3*, *Cxcl1*, *Thbs1*, *Ccnd1,* as well as *Stat3*, were also elevated in *Gfap*^+/R236H^ mice (Figure 1D). Immunofluorescence labeling demonstrated nuclear localization of activated pSTAT3 specifically in *Gfap*^+/R236H^ mouse astrocytes in both forebrain and hindbrain regions (Figure 1E). Importantly, activation of STAT3 was also apparent in brain tissue from a patient with AxD, with nuclear localization of pSTAT3 in astrocytes (Figure 1F). These results suggest that STAT3 contributes to astrocyte reactivity in AxD, but whether the transcription factor is necessary for GFAP accumulation is not clear.

### 3.2. Genetic Knockdown of STAT3 in AxD Model Mice

**Figure 2:**
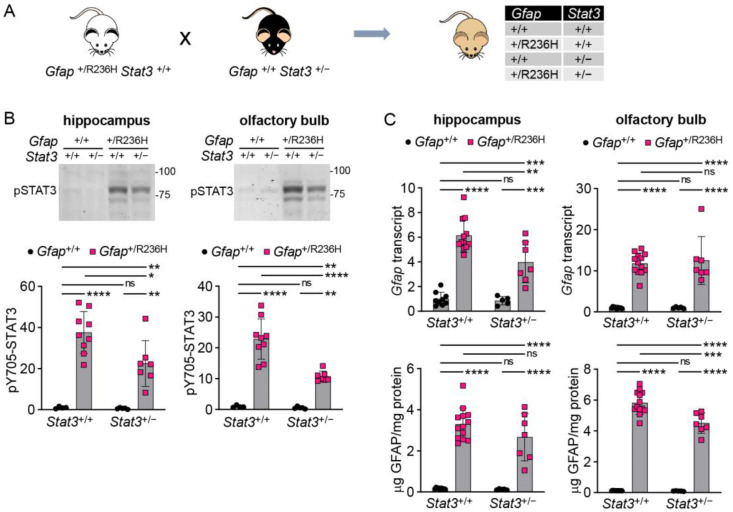
Genetic knockdown of *Stat3* in AxD model mice has marginal effects on *Gfap* expression. (**A**) Genetic cross to generate *Gfap*^+/R236H^ mice with reduced STAT3 activation. (**B**,**C**) Quantitation of pY705-STAT3 by immunoblotting (**B**), and *Gfap* transcript by qPCR and protein by ELISA (**C**) in olfactory bulb and hippocampus from *Gfap*^+/R236H^ mice and *Gfap*^+/+^ littermate controls with heterozygous knockout of the STAT3-Y705 phosphorylation site. Values represent fold change relative to the average for *Gfap*^+/+^*; Stat3*^+/+^ mice. FVBB6F1 male and female mice were used at 12 weeks of age. Two-way ANOVA with Sidak’s post-tests, * *p* < 0.05, ** *p* < 0.01, *** *p* < 0.001, **** *p* < 0.0001.

STAT3 is a pleiotropic transcription factor, and complete knockout by deletion of both alleles is lethal [28]. To further explore whether reduced STAT3 activation affects GFAP expression, we generated heterozygous constitutive knockout mice from the *Stat3*^tm2Aki^ strain [22] (see methods), and this line was crossed with *Gfap*^+/R236H^ mice (Figure 2A). At 12 weeks of age, *Gfap*^+/R236H^; *Stat3*^+/−^ mice exhibited reduced pSTAT3 protein in hippocampus and olfactory bulb (Figure 2B), two regions known to have elevated GFAP in AxD mouse models. Quantitation of *Gfap* transcript and GFAP protein demonstrated only subtle and inconsistent differences (Figure 2C), suggesting that even at reduced levels, pSTAT3 activation is still sufficient to elevate *Gfap* expression, or that other pathways are involved in *Gfap* transactivation in AxD.

### 3.3. Conditional Knockout of STAT3 in Gfap Expressing Cells Prevents GFAP Elevation and Stress Response in AxD Model Mice

**Figure 3:**
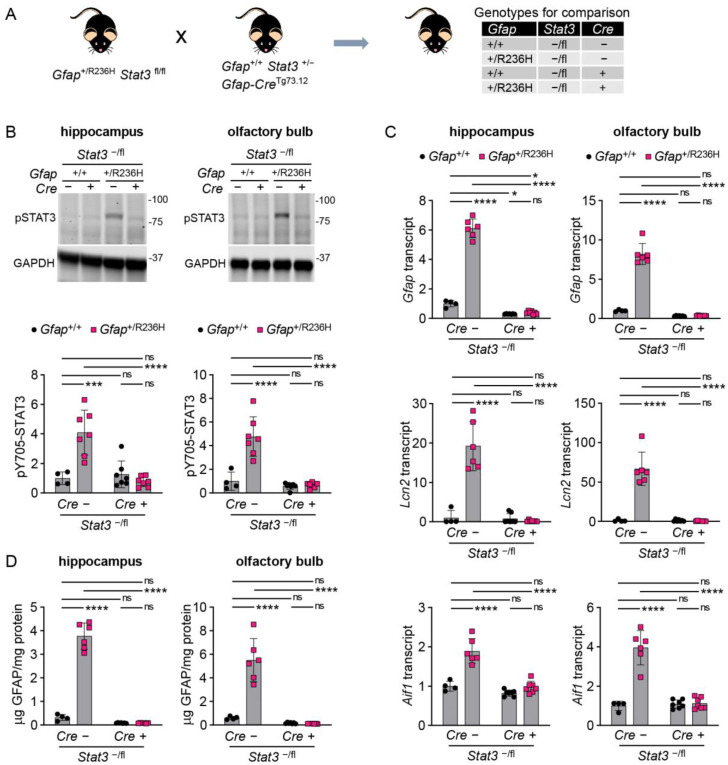
GFAP and stress markers in AxD model mice with conditional genetic knockout of *Stat3* in *Gfap* expressing cells at 8 weeks of age. (**A**) Genetic cross to generate *Gfap*^+/R236H^ mice and *Gfap*^+/+^ littermate controls with heterozygous knockout/floxed (-/fl) *Stat3* alleles and a *Gfap*-promoter-driven Cre transgene Tg73.12. (**B**–**D**) Quantitation of pY705-STAT3 protein by immunoblotting (**B**), *Gfap*, *Lcn2*, and *Aif1* transcripts by qPCR (**C**), and GFAP protein by ELISA (**D**) in hippocampus and olfactory bulb. Values in (**B**,**C**) are normalized to GAPDH and *Rn18s*, respectively, and represent fold change relative to the average for *Gfap*^+/+^; *Stat3*^−^^/fl^; Cre (-) mice. C57BL/6J males and females, two-way ANOVA with Sidak’s post-tests, * *p* < 0.05, *** *p* < 0.001, **** *p* < 0.0001, ns = not significant.

To specifically assess the role of STAT3 in *Gfap* expressing cells, we next used a transgenic line (Tg73.12) expressing Cre recombinase under the control of the mouse *Gfap* promoter [23], in combination with the floxed (fl) *Stat3* mice. The genes for GFAP and STAT3 are located within a 2 megabase interval on mouse chromosome 11. However, we were successful in combining the floxed *Stat3* allele with the R236H *Gfap* mutation, which required a homologous recombination event to generate a *Gfap*^+/R236H^; *Stat3*^fl/fl^ line. To facilitate complete STAT3 knockout in astrocytes, the *Gfap*-Cre-Tg73.12 line was crossed with heterozygous *Stat3*^+/−^ knockout mice to produce Tg73.12; *Stat3*^+/−^ mice. This line was crossed with the *Gfap*^+/R236H^; *Stat3*^fl/fl^ line, and animals carrying both knockout and floxed *Stat3* alleles (-/fl) were analyzed (Figure 3A, all mice congenic in C57BL/6J). At 8 weeks of age *Gfap*^+/R236H^; *Stat3*^−/fl^ mice negative for the Tg73.12 Cre transgene showed the typical increase in pSTAT3 activation, while those that were positive for Cre demonstrated pSTAT3 levels similar to mice with wild-type *Gfap* in both hippocampus and olfactory bulb (Figure 3B). Analysis of *Gfap* mRNA in the same mice demonstrated that conditional *Stat3* knockout in astrocytes reduced transcription to levels at or below the amount observed in *Gfap*^+/+^ mice (Figure 3C). Quantification of *Lcn2* (lipocalin 2) and *Aif1* (Iba1) as markers of reactive astrocytes and microglia, respectively, also showed normalization in *Gfap*^+/R236H^ mice with *Stat3* knockout (Figure 3C). Finally, GFAP protein measures showed reduction with *Stat3* knockout in *Gfap*^+/R236H^ mice (Figure 3D), suggesting that protein accumulation is tied to elevated expression.

**Figure 4:**
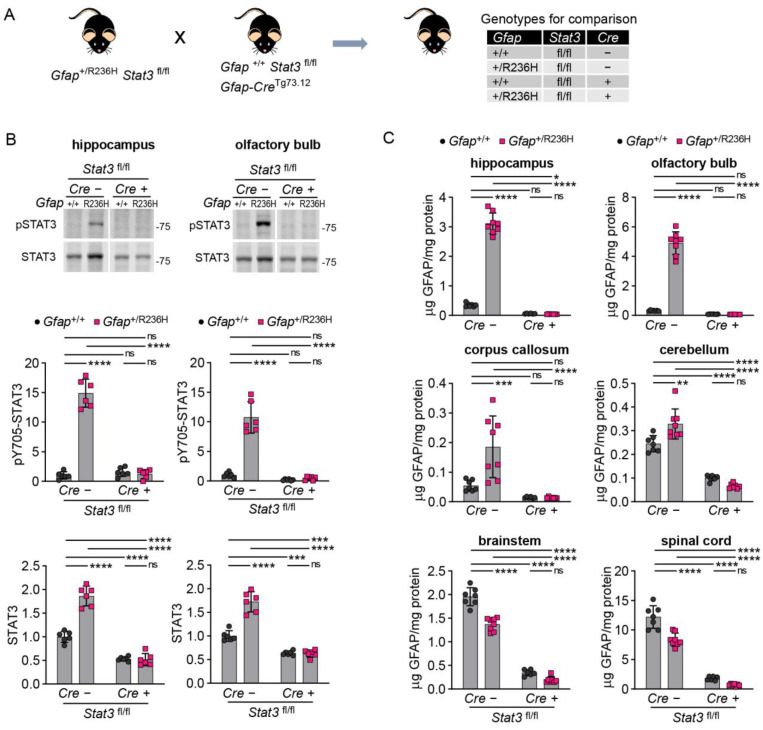
GFAP protein in AxD model mice with conditional genetic knockout of *Stat3* in *Gfap* expressing cells at 12 weeks of age. (**A**) Genetic cross to generate *Gfap*^+/R236H^ mice and *Gfap*^+/+^ littermate controls with both *Stat3* alleles floxed (fl/fl) and a *Gfap*-promoter-driven Cre transgene (Tg73.12). (**B**,**C**) Quantitation of pY705-STAT3 and total STAT3 protein in olfactory bulb and hippocampus by immunoblotting (**B**), and GFAP protein in multiple brain regions and cervical spinal cord by ELISA (**C**). Values in (**B**) are normalized to total protein and represent fold change relative to the average for *Gfap*^+/+^*; Stat3*^fl/fl^; Cre (-) mice. C57BL/6J males and females, two-way ANOVA with Sidak’s post-tests, * *p* < 0.05, ** *p* < 0.01, *** *p* < 0.001, **** *p* < 0.0001, ns = not significant.

To assess whether GFAP mutation might eventually cause protein accumulation with age, we performed similar experiments in older mice (12 weeks and 6 months), when pathology was increased in the model. For these experiments, we used mice carrying the floxed *Stat3* gene on both alleles (fl/fl) for a more exact comparison between full STAT3 expression and the targeted knockout condition (Figure 4A). At 12 weeks of age, we observed a reduction in phosphorylated STAT3 similar to that observed above at 8 weeks, and total STAT3 expression, which was also elevated in *Gfap*^+/R236H^ mice, was normalized by blocking STAT3 activation (Figure 4B). GFAP protein was reduced by STAT3 inactivation in all brain regions analyzed and spinal cord (Figure 4C), with no significant difference between Cre positive *Gfap*^+/R236H^ and *Gfap*^+/+^ mice. Complete knockout of *Stat3* resulted in GFAP levels at or below those measured in wild-type animals.

**Figure 5:**
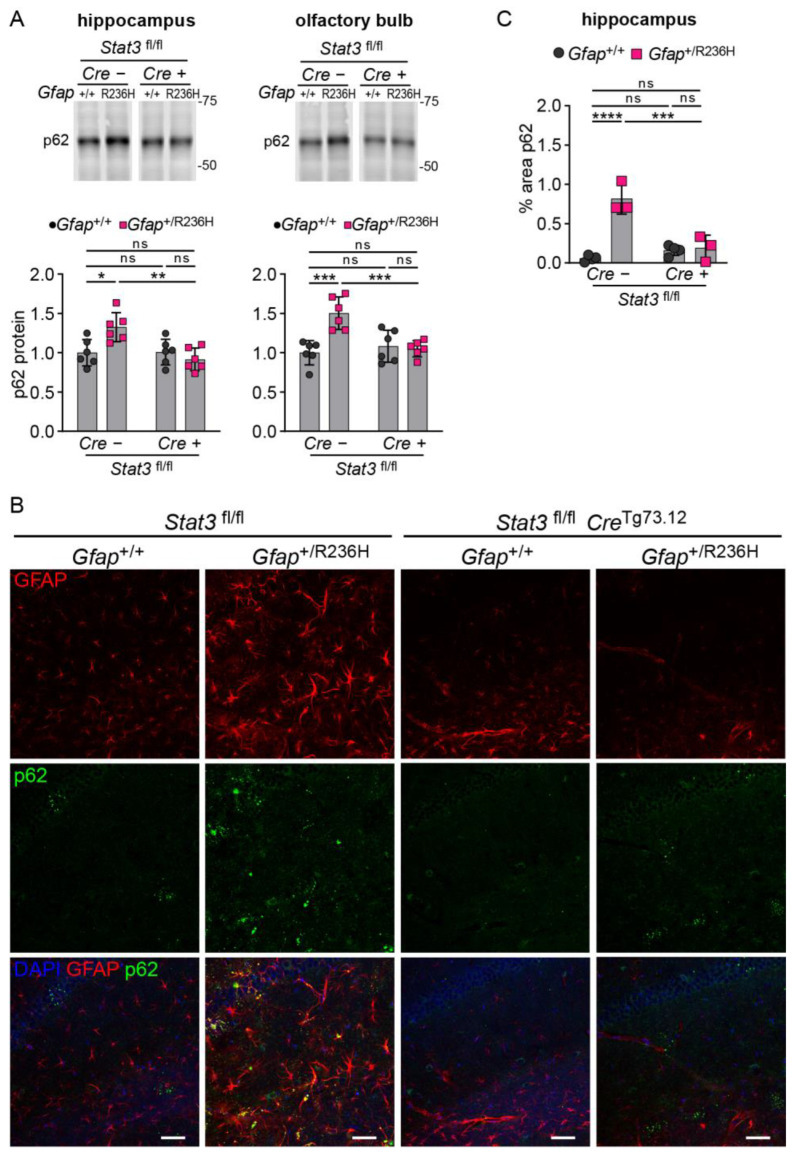
p62/SQSTM1 expression and protein aggregation in AxD model mice with conditional knockout of *Stat3* in *Gfap* expressing cells. (**A**) Quantification of p62/SQSTM1 protein in olfactory bulb and hippocampus from *Gfap*^+/R236H^ mice and *Gfap*^+/+^ littermate controls (3 months of age) collected from the same Stat3^fl/lf^; Cre^Tg73.12^ genetic cross as shown in Figure 4A. (**B**,**C**) Immunofluorescence labeling of p62 and GFAP in hippocampus (**B**), and quantification of percent area for p62 (**C**) in mice from the same cross at 6 months of age. Values in (**A**) are normalized to total protein and represent fold change relative to the average for *Gfap*^+/+^; *Stat3*^fl/fl^; Cre (-) mice. C57BL/6J males and females, two-way ANOVA with Sidak’s post-tests, * *p* < 0.05, ** *p* < 0.01, *** *p* < 0.001, **** *p* < 0.0001, ns = not significant. Scale bars = 50 µm.

**Figure 6:**
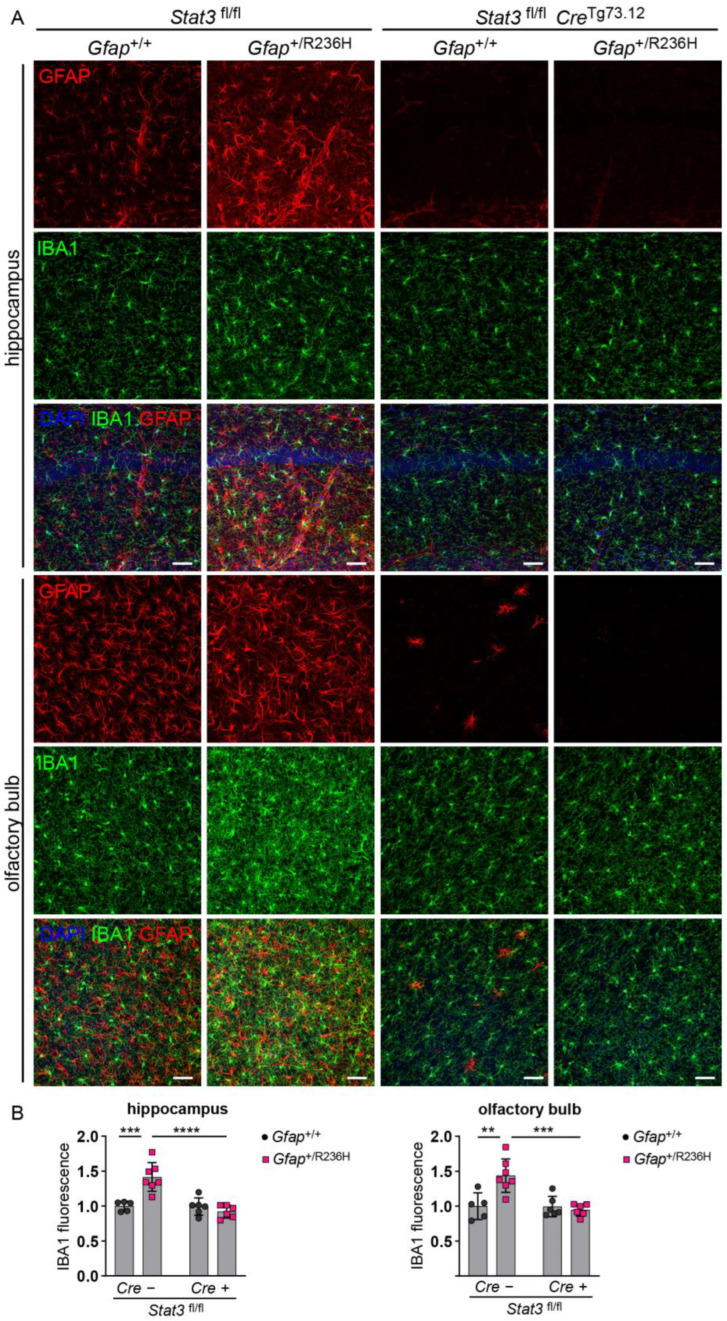
Iba1 expression in microglia from AxD model mice with conditional knockout of *Stat3* in *Gfap* expressing cells. (**A**,**B**) Immunofluorescence labeling (**A**) and quantification (**B**) of Iba1 in hippocampus and olfactory bulb from *Gfap*^+/R236H^ mice and *Gfap*^+/+^ littermate controls (6 months of age) collected from the same Stat3^fl/lf^, Cre^Tg73.12^ genetic cross as shown in Figure 4A. GFAP is also labeled for comparison. Values in (**B**) represent fold change relative to the average for *Gfap*^+/+^; *Stat3*^fl/fl^; Cre (-) mice. C57BL/6J males and females, two-way ANOVA with Sidak’s post-tests, ** *p* < 0.01, *** *p* < 0.001, **** *p* < 0.0001, ns = not significant. Scale bars = 50 µm.

To assess disrupted proteostasis and protein aggregation, we analyzed the autophagy cargo protein p62/SQSTM1 (sequestosome), which facilitates degradation of ubiquitinated proteins and colocalizes with Rosenthal fibers [29,30], and found that blocking STAT3 activation reduced p62 accumulation in *Gfap*^+/R236H^ mice as shown by western analysis in Figure 5A. These results were confirmed by immunofluorescence in animals at 6 months of age, which exhibited no GFAP aggregation or p62 accumulation when STAT3 phosphorylation was prevented in astrocytes (Figure 5B,C). Finally, Iba1 immunolabeling showed reduced microglial expression, indicating the prevention of neuroinflammation (Figure 6).

### 3.4. Inducible STAT3 Knockout in Adult AxD Mouse Model Astrocytes Reverses GFAP Pathology and Normalizes Reactive Astrocytes and Microglia

**Figure 7:**
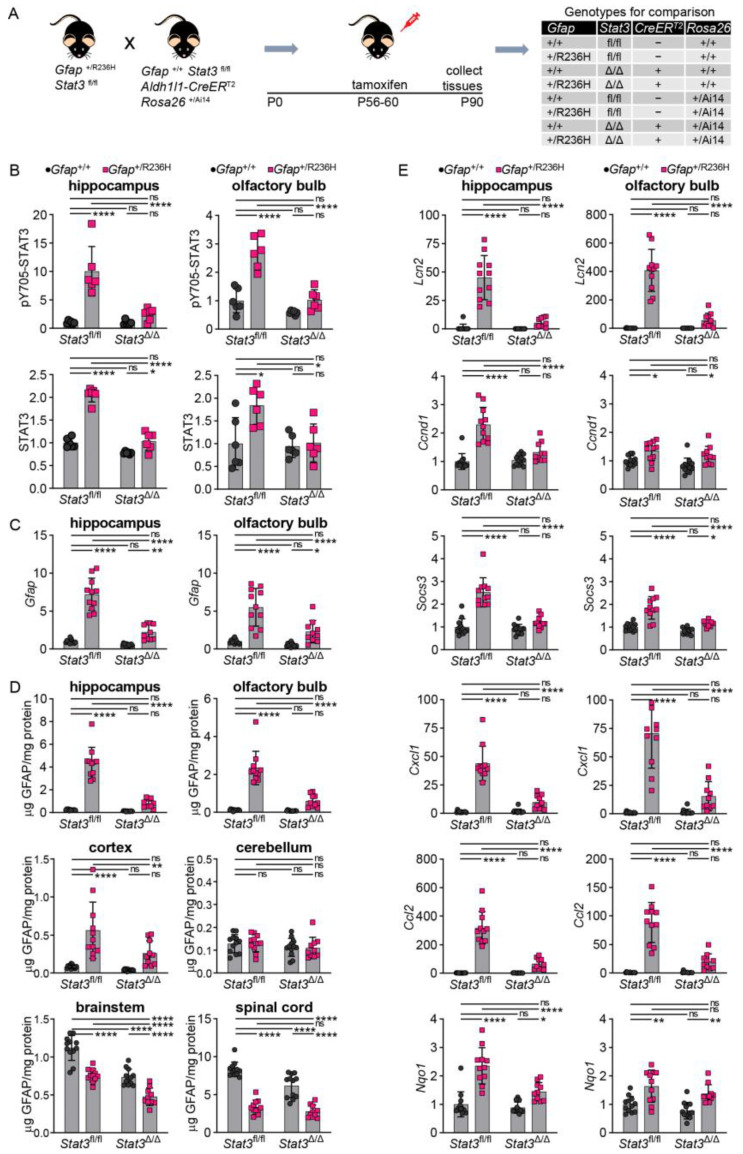
STAT3, GFAP, and other stress and immune related markers in AxD model mice after induced genetic knockout of *Stat3* in astrocytes. (**A**) Genetic cross to generate *Gfap*^+/R236H^ mice and *Gfap*^+/+^ littermate controls with both *Stat3* alleles floxed (fl/fl) and a tamoxifen-inducible *Aldh1l1*-promoter driven CreER^T2^ transgene. All mice were treated with tamoxifen at 2 months of age for 5 days, and tissues collected at 3 months of age. Timeline in right panel shows targeted postnatal (P) days. *Stat3* alleles in animals expressing the *Aldh1l1*-CreER^T2^ transgene are indicated as Δ (Stat3^Δ/Δ^). (**B**,**C**) Quantitation of pY705-STAT3 and total STAT3 protein by immunoblotting (**B**), and *Gfap* transcript by qPCR (**C**) in hippocampus and olfactory bulb. (**D**) GFAP protein quantification in multiple brain regions and cervical spinal cord. (**E**) Quantification of transcripts reflecting reactive gliosis (*Lcn2*), STAT3 activation (*Ccnd1*, *Socs3*), neuroinflammation (*Cxcl1*, *Ccl2*), and oxidative stress (*Nqo1*) in hippocampus and olfactory bulb. Values in (**B**) are normalized to total protein and in (**C**,**E**) to *Rn18s*, and all represent fold change relative to the average for *Gfap*^+/+^; *Stat3*^fl/fl^; Cre (-) mice. C57BL/6J males and females, two-way ANOVA with Sidak’s post-tests, * *p* < 0.05, ** *p* < 0.01, **** *p* < 0.0001, ns = not significant.

To investigate whether genetic manipulation of STAT3 phosphorylation can affect GFAP accumulation in adult AxD model mice with established pathology [5,6], we took advantage of a tamoxifen-inducible astrocyte-specific CreER^T2^ line using the *Aldh1l1* promoter [24]. We first crossed the *Aldh1l1*-CreER^T2^ line to the Cre-reporter line Ai14 [25] and subsequently bred those mice with the Stat3^fl/fl^ line. *Gfap*^+/R236H^; *Stat3*^fl/fl^ mice were then crossed to the CreER^T2^; Ai14; *Stat3*^fl/fl^ line. *Gfap*^+/R236H^ and *Gfap*^+/+^ animals that were either positive or negative for Cre were treated with tamoxifen for 5 days at 2 months of age, and tissues collected at 3 months of age (Figure 7A).

An initial assessment of efficacy for tamoxifen induction of Cre recombination in astrocytes using the Ai14 tdTomato reporter showed that 83% of GFAP positive astrocytes in hippocampus and 87% in olfactory bulb were also positive for tdTomato (364+/436 and 748+/858 astrocytes, respectively). We determined whether STAT3 was activated and found that *Gfap*^+/R236H^; *Stat3*^fl/fl^ mice (without Cre) showed the same increase in pSTAT3, whereas mice expressing Cre (*Stat3*^Δ/Δ^) demonstrated pSTAT3 levels similar to those of *Gfap*^+/+^ mice in both hippocampus and olfactory bulb (Figure 7B). Total STAT3 protein showed similar changes, although not as pronounced.

We next examined whether blocking STAT3 activation in adult AxD model mice could reverse GFAP accumulation. Transcript analysis demonstrated a significant reduction in *Gfap* expression in hippocampus and olfactory bulb (Figure 7C). Analysis of GFAP protein showed reductions in all brain regions examined except for cerebellum (Figure 7D). Spinal cord, which had decreased amounts of GFAP in *Gfap*^+/R236H^ mice, also showed no further reduction in protein. *Lcn2* transcript was measured as a surrogate marker of astrogliosis, and expression was normalized with STAT3 inactivation (Figure 7E). Other transcripts regulated by STAT3, including *Socs3* and *Ccnd1*, were also decreased in hippocampus, and *Socs3* was reduced in olfactory bulb. Transcripts for small chemokines *Cxcl1* and *Ccl2* were reduced, suggesting a normalization of the neuroinflammatory response, and *Nqo1* (NAD(P)H quinone oxidoreductase), a target of the Nrf2/ARE antioxidant stress response pathway, was reduced in hippocampus but not olfactory bulb (Figure 7E).

**Figure 8:**
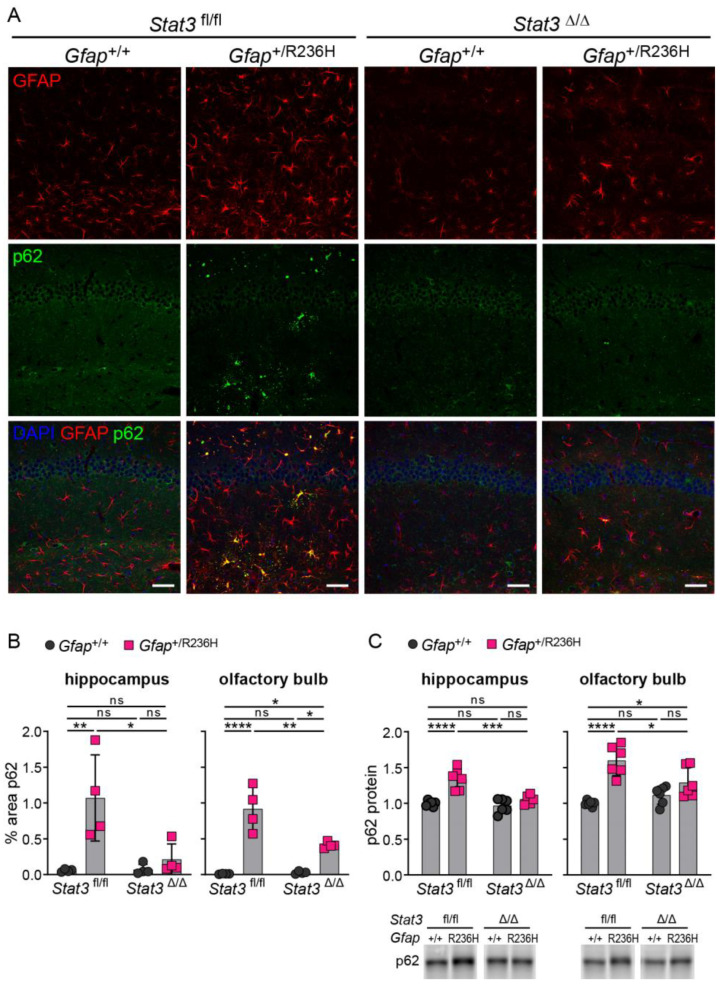
p62/SQSTM1 expression and protein aggregation in AxD model mice after inducing genetic knockout of *Stat3* in astrocytes. (**A**,**B**) Immunofluorescence labeling of p62 and GFAP in hippocampus (**A**), and quantitation of percent area (**B**) in hippocampus and olfactory bulb from *Gfap*^+/R236H^ mice and *Gfap*^+/+^ littermate controls (3 months of age) derived from the same Stat3^fl/fl^, CreER^T2^ genetic cross and tamoxifen treatment schedule as shown in Figure 7A. *Stat3* alleles in animals expressing the *Aldh1l1*-CreER^T2^ transgene are indicated as Δ (Stat3^Δ/Δ^). (**C**) Quantification of p62/SQSTM1 protein in olfactory bulb and hippocampus by western analysis of a separate group of mice from the same genetic cross and treatment described in (**A**,**B**). C57BL/6J males and females, two-way ANOVA with Sidak’s post-tests, * *p* < 0.05, ** *p* < 0.01, *** *p* < 0.001, **** *p* < 0.0001, ns = not significant. Scale bars = 50 µm.

To determine whether reduced GFAP expression led to reduced aggregation and improved proteostasis, we immunolabeled p62 positive aggregates as a measure of Rosenthal fibers and found that protein aggregation was reversed in *Gfap*^+/R236H^ mice by blocking STAT3 activation (Figure 8A,B). Quantification of p62 by western analysis showed a similar reduction in hippocampus and olfactory bulb (Figure 8C).

**Figure 9:**
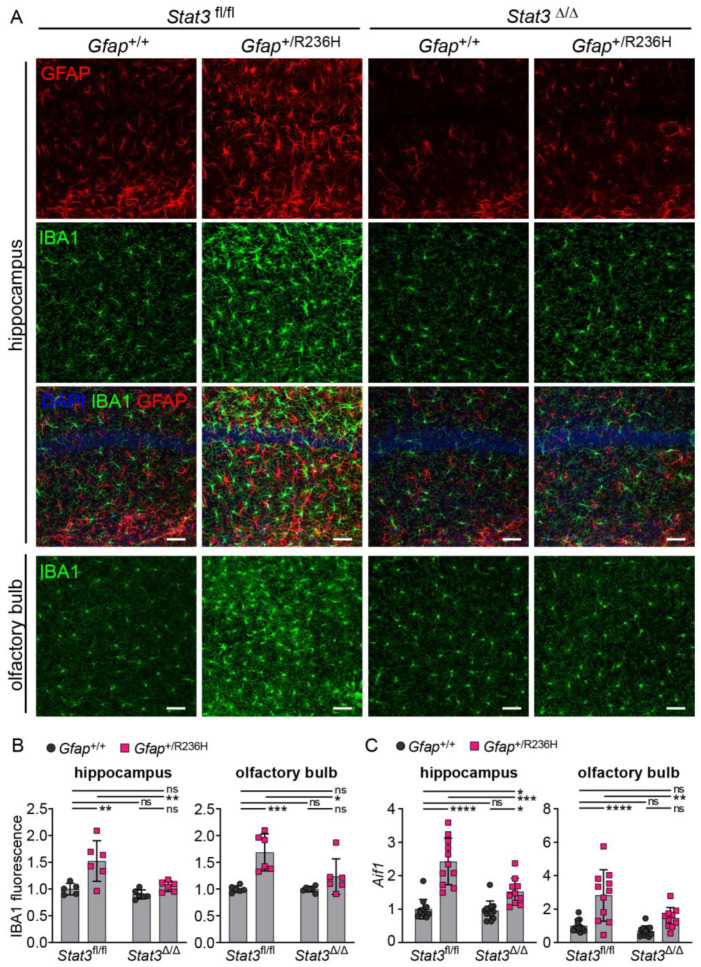
Microglial Iba1 expression in AxD model mice after inducible knockout of *Stat3* in astrocytes. (**A**,**B**) Immunofluorescence labeling (**A**) and quantification (**B**) of Iba1 in hippocampus and olfactory bulb from *Gfap*^+/R236H^ mice and *Gfap*^+/+^ littermate controls (3 months of age) derived from the same *Stat3*^fl/fl^, CreER^T2^ genetic cross and tamoxifen treatment schedule as shown in Figure 7A. *Stat3* alleles in animals expressing the *Aldh1l1*-CreER^T2^ transgene are indicated as Δ (Stat3^Δ/Δ^). GFAP is also shown in hippocampus for comparison. (**C**) Quantification of *Aif1* transcript (Iba1 gene, normalized to *Rn18s*) in olfactory bulb and hippocampus by qPCR. Values represent fold change relative to the average for *Gfap*^+/+^; *Stat3*^fl/fl^; Cre (-) mice. C57BL/6J males and females, two-way ANOVA with Sidak’s post-tests, * *p* < 0.05, ** *p* < 0.01, *** *p* < 0.001, **** *p* < 0.0001, ns = not significant. Scale bars = 50 µm.

Finally, to assess the effects of STAT3 inhibition in astrocytes on neuroinflammation in general, we examined microglia activation by labeling with Iba1 and found that microglia appeared to return to a normal state with reduced Iba1 expression (Figure 9A,B). Transcript analysis of the gene for Iba1 (*Aif1*) also showed a reduction in hippocampus and olfactory bulb after blocking STAT3 activation in *Gfap*^+/R236H^ mice (Figure 9C).

## 4. Discussion

GFAP expression and accumulation have been shown to correlate with AxD severity [8], and STAT3 is known to regulate GFAP transcription in development and in response to injury [14]. In mouse models of AxD, regions of the CNS with the greatest increase in GFAP expression also demonstrate significant STAT3 phosphorylation. In addition, we showed that STAT3 was activated in gray matter astrocytes in a patient with the disease. Here, we investigated whether blocking STAT3 phosphorylation could be used to manipulate *Gfap* expression and prevent or reverse protein accumulation in *Gfap*^+R236H^ mice. Initial experiments with heterozygous *Stat3*^+/−^ knockout mice achieved significant reduction in phosphorylated STAT3-Y705, but these differences were not sufficient to consistently reduce *Gfap* expression. In addition to STAT3, several other transcription factors and their binding sites in the *GFAP* promoter have been tested for their influence on basal expression and the injury response [14]. AP-1 in particular has been suggested to regulate *Gfap* elevation in different injury models [31], including a transgenic mouse model of AxD that overexpresses wild-type human GFAP [7], but whether AP-1 or other factors contribute to *Gfap* elevation in *Gfap*^+R236H^ mice has not yet been tested.

To further investigate the role of STAT3 in reactive AxD astrocytes, we used a conditional knockout approach to target *Stat3* in *Gfap* expressing cells and found that *Stat3* knockout reduced GFAP levels and prevented the reactive astrocyte response, similarly to other studies in acute injury models [12,15] and models of neurodegeneration [16,32]. STAT3 is an essential regulator of astrocyte differentiation and maturation, and by prohibiting STAT3 activation during astrocyte development, GFAP transcript and protein were reduced even in *Gfap*^+/+^ mice. AxD severity is tied to GFAP expression, and in these experiments, further transactivation of the *Gfap* promoter was prevented. Reduced GFAP suggests that mutant protein does not reach a toxic threshold and that astrocytes are able to maintain proteostasis and avoid a reactive stress response.

GFAP is also expressed in progenitor cells, and early activation of the *Gfap*-promoter-driven Cre transgene (Tg73.12) as described above leads to excision of floxed genes in both astrocytes and other differentiated cell types, including neurons [23]. To specifically evaluate the role of STAT3 activation in reactive astrocytes with existing GFAP accumulation and protein aggregation [5,6], we used an inducible CreER^T2^ line controlled by the *Aldh1l1* promoter [24]. Knocking down STAT3 in adult mice with established GFAP pathology reduced *Gfap* expression and the concomitant stress and neuroinflammatory response and reversed GFAP accumulation and aggregation. We cannot exclude the formal possibility that Cre itself has an effect independent of STAT3 [33]; however, we consider this unlikely given the lack of change in markers for astro- and microgliosis (e.g., *Lcn2*, *Aif1*) in the presence of Cre in *Gfap*^+/+^ mice (Figure 3C, Figure 7E and Figure 9C). These results demonstrate that STAT3 is a key factor in *Gfap* elevation and GFAP accumulation, and that blocking STAT3 activation may provide a therapeutic target for AxD.

STAT3 activation is not necessarily detrimental, and as has been shown in other models of CNS injury or neurodegenerative disease, the consequences are context-dependent [34,35,36,37,38,39,40]. In Huntington’s disease, for example, activation of JAK/STAT3 signaling improves proteostasis by increasing gene expression in proteasome and lysosomal pathways [39]. However, given the role of STAT3 in *Gfap* transactivation, inhibiting STAT3 in AxD would have the direct benefit of reducing levels of mutant GFAP and preventing protein misfolding and the resulting cellular toxicity. More extensive analyses of AxD tissues and controls will be needed to firmly establish the role of STAT3 in the human disorder.

Although we do not know the initiating events leading to STAT3 activation, our results support our hypothesis that activation of STAT3 contributes to a positive feedback loop that drives increased GFAP expression, protein accumulation, and ultimately astrocyte dysfunction. GFAP is one of the most highly expressed proteins in astrocytes, and a misfolded mutant form could disrupt a myriad of cellular processes, including vesicular transport and mitochondrial function, which could lead to oxidative stress and activation of MAP kinase pathways. Activation of JNK and p38 has been demonstrated in cellular models of AxD [41,42], and MAP kinases could activate AP-1 and NFκB, leading to increased IL-6 to activate the IL-6/JAK/STAT3 pathway.

In this study, we took advantage of the wealth of available genetic tools to manipulate STAT3 activation in our mouse models of AxD. *Gfap*^+R236H^ mice recapitulate many of the pathological features of human disease and have been instrumental in investigating mechanisms and modifiers of the disease [43]; however, they have only subtle behavioral deficits and limited functional outcome measures [5,44]. For future studies to test whether inhibiting STAT3 pharmacologically has therapeutic value, we recently developed a rat model of AxD [19], which, in addition to STAT3 activation, exhibits motor deficits and other clinically relevant phenotypes for improved functional assessment. Inhibitors of the JAK/STAT pathway have been approved to treat inflammatory disorders, hematopoietic malignancies, and solid tumors [20,21], and JAK inhibitors have been shown to be effective in suppressing STAT3 activation in glioblastoma, experimental autoimmune encephalomyelitis (EAE) models of multiple sclerosis, and a model of Parkinson’s disease [45,46,47,48]. The JAK inhibitor baricitinib was recently evaluated in clinical trials for Aicardi–Goutieres syndrome, an interferonopathy that also leads to white matter deficits, with results suggesting improved neurological function [49]. Although JAK inhibition can have adverse side effects [50,51], strategies to specifically target STAT3 with small molecules have been effective in preclinical models of Alzheimer’s disease [32,52] and may provide alternatives for reducing GFAP pathology in AxD.

## Figures and Tables

**Table 1:** Primer sets for quantitative PCR.

Gene	Forward Primer	Reverse Primer
*Gfap*	CAA CGT TAA GCT AGC CCT GGA CAT	CTC ACC ATC CCG CAT CTC CAC AGT
*Aif1*	GCC AAA GCA GGG ATT TGC AG	GGG AAC CCC AAG TTT CTC CA
*Ccl2*	CTG GAG CAT CCA CGT GTT GG	CAT TCC TTC TTG GGG TCA GC
*Ccnd1*	GCG TAC CCT GAC ACC AAT CTC CT	CTT CGC ACT TCT GCT CCT CAC
*Cxcl1*	GCC ACA CTC AAG AAT GGT CG	ACC AGA CAG GTG CCA TCA GA
*Il6*	GAC TTC CAT CCA GTT GCC TTC T	AAG TAG GGA AGG CCG TGG TT
*Lcn2*	AGA CTT CCG GAG CGA TCA GT	TCT GAT CCA GTA GCG ACA GC
*Nqo1*	CGG TAT TAC GAT CCT CCC TCA ACA	AGC CTC TAC AGC AGC CTC CTT CAT
*Socs3*	AAG GCC GGA GAT TTC GCT TC	GGG AAA CTT GCT GTG GGT GA
*Stat3*	ACG AAA GTC AGG TTG CTG GT	GCT GCC GTT GTT AGA CTC CT
*Thbs1*	TGC CCT GCC CAC CAC GAT TCA	GCA TAG CCG GGC TTG CAC TCA CA
*Rn18s*	CGC CGC TAG AGG TGA AAT TCT	CGA ACC TCC GAC TTT CGT TCT

**Table 2:** Antibodies used in study.

Assay	Antigen	Host	Mono/Poly	Source	Catalog No.	RRID	Dilution
ELISA	GFAP	mouse	mono SMI 26	BioLegend	837602	AB_2565380	1:1000
ELISA	GFAP	rabbit	poly	Dako/Agilent	Z0334	AB_10013382	1:5000
IF	GFAP	mouse	mono GA5	Millipore Sigma	G6171	AB_1840893	1:1000
IF	GFAP	rabbit	poly	Dako/Agilent	Z0334	AB_10013382	1:1000
IF	Iba1	rabbit	poly	Wako	019-19741	AB_839504	1:500
IF	p62/SQSTM1	mouse	mono 2C11	Abnova	H00008878-M01	AB_437085	1:100
IF	STAT3-pY705	rabbit	mono D3A7	Cell Signaling	9145	AB_2491009	1:200
Western	GAPDH	mouse	mono 6C5	Fitzgerald	10R-G109A	AB_1285808	1:10,000
Western	GAPDH	rabbit	poly	Abcam	Ab9485	AB_307275	1:2500
Western	p62/SQSTM1	mouse	mono 2C11	Abnova	H00008878-M01	AB_437085	1:5000
Western	STAT3	mouse	mono 124H6	Cell Signaling	9139S	AB_331757	1:5000
Western	STAT3-pY705	rabbit	mono D3A7	Cell Signaling	9145S	AB_2799407	1:5000
							
**Assay**	**2nd Antigen**	**Host**	**Conjugate**	**Source**	**Catalog No.**	**RRID**	**Dilution**
ELISA	rabbit-IgG	goat	HRP	Millipore Sigma	A6154	AB_258284	1:10,000
IF	mouse-IgG	goat	Alexa Fluor 568	Thermo-Invitrogen	A11031	AB_144696	1:500
IF	mouse-IgG	goat	Alexa Fluor 647	Thermo-Invitrogen	A32728	AB_2633277	1:500
IF	rabbit-IgG	goat	Alexa Fluor 488	Thermo-Invitrogen	A11034	AB_2576217	1:500
IF	mouse-IgG	donkey	Alexa Fluor 488	Thermo-Invitrogen	A21202	AB_141607	1:500
IF	rabbit-IgG	donkey	Alexa Fluor 546	Thermo-Invitrogen	A10040	AB_2534016	1:500
Western	mouse-IgG	goat	Alexa Fluor 680	Thermo-Invitrogen	A21057	AB_2535723	1:10,000
Western	mouse-IgG	goat	DyLight 800	Thermo Scientific	SA5-10176	AB_2556756	1:10,000
Western	rabbit-IgG	goat	Alexa Fluor 680	Thermo-Invitrogen	A21109	AB_2535758	1:10,000
Western	rabbit-IgG	goat	DyLight 800	Thermo Scientific	35571	AB_614947	1:10,000

## Data Availability

All relevant data are included in the current report.
